# Frailty is Linked to a Lower Subjective Life Expectancy Among Korean Middle-Aged and Older Adults

**DOI:** 10.1093/geroni/igaf122.2370

**Published:** 2025-12-31

**Authors:** Juhyeong Lee, Yeonsoo Shin, Giyeon Kim

**Affiliations:** The University of British Columbia, Vancouver, British Columbia, Canada; The University of Texas at Austin, Austin, Texas, United States; Chung-Ang University, Seoul, Republic of Korea

## Abstract

Frailty in later life can lead to an identity crisis, potentially limiting individuals’ perception of their future and opportunities. This study examines differences in subjective life expectancy (SLE) between frail and non-frail Korean middle-aged and older adults. The sample was drawn from the Wave 1 of the Korean Longitudinal Study of Aging (N = 10,254). SLE was assessed based on respondents’ perceived likelihood of surviving for another 10 to 15 years, given their current age. Frailty was measured using 33 items, including self-rated health and chronic disease diagnoses. Participants with a score of 2 or higher were classified as frail (n = 682), and those with a score below 2 as non-frail (n = 8,735). To ensure homogeneous characteristics between the two groups, we performed propensity score matching with 1:1 nearest neighbor matching. A final matched sample of 1,364 participants (M age = 73.5 years, SD = 10.0, range = 45-98; 62% female), with 682 in each group was included in the final analysis, showing no significant demographic differences. Results from a multiple linear regression analysis showed that frailty was significantly associated with lower SLE scores (B = -17.66, p < .001). Additionally, an interaction analysis indicated that the effect of age on SLE varied by frailty status (B = 0.56, p < .001). Findings suggest that frail Korean middle-aged and older adults perceive their life expectancy as lower than their non-frail counterparts. This highlights the importance of addressing frailty in older adults to support positive future outlooks.

